# Skin Cancer Epidemics in the Elderly as An Emerging Issue in Geriatric Oncology

**DOI:** 10.14336/AD.2017.0503

**Published:** 2017-10-01

**Authors:** Simone Garcovich, Giuseppe Colloca, Pietro Sollena, Bellieni Andrea, Lodovico Balducci, William C. Cho, Roberto Bernabei, Ketty Peris

**Affiliations:** ^1^Institute of Dermatology, Policlinico A. Gemelli University Hospital, Catholic University of the Sacred Heart, Rome, Italy; ^2^Department of Geriatrics, Policlinico A. Gemelli University Hospital, Catholic University of Sacred Heart, Rome, Italy.; ^3^Senior Adult Oncology Program, Moffitt Cancer Center, Tampa, FL, USA; ^4^Department of Clinical Oncology, Queen Elizabeth Hospital, Kowloon, Hong Kong

**Keywords:** skin cancer, elderly cancer patients, geriatrics, basal cell carcinoma, squamous cell carcinoma, melanoma, geriatric assessment, disease management

## Abstract

Skin cancer is a worldwide, emerging clinical need in the elderly white population, with a steady increase in incidence rates, morbidity and related medical costs. Skin cancer is a heterogeneous group of cancers comprising cutaneous melanoma and non-melanoma skin cancers (NMSC), which predominantly affect elderly patients, aged older than 65 years. Melanoma has distinct clinical presentations in the elderly patient and represents a challenging question in terms of clinical management. NMSC includes the basal cell carcinoma and cutaneous squamous cell carcinoma and presents a wide disease spectrum in the elderly population, ranging from low-risk to high-risk tumours, advanced and inoperable disease. Treatment decisions for NMSC are preferentially based on tumour characteristics, patient’s chronological age and physician’s preferences and operational settings. Several treatment options are available for NMSC, from surgery to non-invasive/medical therapies, but patient-based factors, such as geriatric comorbidities and patient’s life expectancy, do not frequently modulate treatment goals. In melanoma, age-related variations in clinical management are significant and may frequently lead to under-treatment, limiting access to advanced surgical and medical treatments. Clinical decision-making in the care of elderly skin cancer patient should ideally implement a geriatric assessment, prioritizing patient-based factors and efficiently differentiating fit from frail cancer patients. Current clinical practice guidelines for NMSC and melanoma only partially address geriatric aspects of cancer care, such as frailty, limited life-expectancy, geriatric comorbidities and treatment compliance. We review the recent evidence on the scope and problem of skin cancer in the elderly population as well as age-related variations in its clinical management, highlighting the potential role of a geriatric approach in optimizing dermato-oncological care.

The worldwide surge in the incidence of skin cancer during the last two decades has reached “epidemic” proportions, resulting from long, lifetime sun exposure in an increasingly aging population [1]. Skin cancer significantly contributes to the overall burden of cutaneous conditions in the elderly population, determining significant morbidity, mortality and health-related costs.

**Table 1:** Prevalence rates of skin cancer and precursors in geriatric populations according to study setting.

Study setting	Skin cancer	Prevalence-rate (%)
	Pre-malignant skin lesions and AKs	10.4-69.4
	All malignant skin cancer	2-12
	BCC	2.8
	cSCC	0.2
	Melanoma	0.1
**Institutional long-term care/nursing homes**	Pre-malignant skin lesions and AKs	4.6-29.3
	All malignant skin cancer	1-5.6
	BCC	3.9-14.8%
	cSCC	8%
	Melanoma	2.3%
**Hospital-based geriatric units**	Pre-malignant skin lesions and AKs	32.8%
	All malignant skin cancer	4.9%
	BCC	-
	cSCC	-
	Melanoma	-
**Hospital/outpatient-based dermatology setting**	Pre-malignant skin cancer and AKs	0.5-39%
	All malignant skin cancer	2-13.2%
	BCC	11-21%
	cSCC	2%
	Melanoma	4%

AKs=actinic keratoses; BCC=Basal cell carcinoma; cSCC=cutaneous squamous cell carcinoma

Skin cancer comprehends two main types of tumours, cutaneous melanoma (CM) and the keratinocytic-epithelial tumours, commonly defined as non-melanoma skin cancers (NMSC), encompassing a heterogeneous clinical spectrum in terms of morbidity and mortality. Several types of tumours fall within the broader category of NMSC, but basal cell carcinoma (BCC) and cutaneous squamous cell carcinoma (cSCC) are the most important from an epidemiological and clinical perspective. NMSC account for at least 80% of all skin cancer cases, with a large prevalence of BCC (70%) over cSCC (20%) in the general population. In 2012, an estimated 3,315,554 Americans were treated for a NMSC, with a significant 35% increase of incidence rates in the US over the preceding 6-year period [2]. NMSC thus has the highest incidence of all cancers, outweighing all other cases of human cancers combined. Skin cancer is associated with a substantial health and economic burden, as it is among the costliest cancers to treat in the US. Average annual total cost for skin cancer increased by 126.2% during the 2007-2011 period, compared to a 25.1% increase for all other cancers, reaching a total of 8.1 billion dollar yearly costs [3]. Annual treatment costs for treating newly diagnosed melanomas are also expected to rise dramatically from 457 million dollars (2011) to 1.6 billion dollars in 2030, due to rising incidence rates, aging population, enduring risk behaviours and development of new targeted-therapies [4]. The white-skinned elderly population thus represent the largest patient group at-risk for developing skin cancer. The definition of an elderly individuals on the basis of pure “chronological” age is difficult, as most studies use variable cut-offs (65, 70 or 75 years). The National Institute on Aging classify elderly persons into young-old (aged 65-75), old (aged 76-85) and oldest-old (older than 85 years), but there is no general agreement on the age at which a person becomes old.

Comprehensive, high-quality epidemiological data on the impact of skin cancer in the elderly population, and respective three age subgroups, is lacking, deriving mostly from retrospective and cross-sectional studies in institutional long care or outpatient settings. The prevalence of skin cancer in the geriatric population has been estimated in 2.1-8.3% in acute o chronic geriatric units or nursing homes, as opposed to higher figures (9-12%) reported in cohorts of elderly patients attending dermatology clinics [5-9]. This disparity in the prevalence of skin cancer in the elderly could be attributed to a selection bias, due to different study designs, case-definitions and geographical origin of published studies (table 1). Systematic skin examination is not part of the comprehensive geriatric assessment, and the presence of skin cancer and suspicious lesions is not routinely recorded (table 2). Diagnosis of skin cancer in specialized care relies increasingly in non-invasive methods, such as dermoscopy, and access of elderly, institutionalized patients to dermatology consultation is limited due to socio-economic barriers. Furthermore, the reported incidence of the NMSC in the general population is largely underestimated, as these tumours are not recorded in national cancer registries. Few studies have reported the association between the diagnosis of skin cancer and the presence of the frailty condition in elderly patients. Dependency, malnutrition, cognitive impairment and other aspects of the frailty syndrome have not been systemically reported in epidemiological and clinical studies on skin cancers in the elderly population. Age-related variations in the clinical management of skin cancer are considerable and cause a significant risk of both over- and under-treatment in the aged population. In the present review, we will describe the clinical burden of the most relevant skin cancers, BCC, CM, in the elderly patients, highlighting the age-related variations and the implications of an oncogeriatric approach in its clinical management.

**Table 2:** Geriatric instruments for an appropriate onco-geriatric assessment.

Questions	Onco-Geriatric Assessment Instrument
Is the patient self sufficient?	ADL, IADL
Has the patient a cognitive impairment?	MMSE
How are the Physical Performance?	SPPB, TUP
Compliance and needs?	InterRAI suite
Is there a Social Network able to protect the patient?	InterRAI suite
How to calculate the prognostic value of biological age?	Active Life Expectancy

## Methods

We conducted a literature search in the relevant databases (PUBMED, EMBASE, Web of Science), employing the following keywords and their respective combinations: “elderly”, “older patient”, “skin cancer”, “non-melanoma skin cancer”, “basal cell carcinoma”, “cutaneous squamous cell carcinoma”, “cutaneous melanoma”, “frailty”, “oncogeriatric assessment”, “oncogeriatric intervention”, “quality of life”, “life expectancy”, “treatment of skin cancer”. Relevant published studies and reports from 1996 onwards were included. We reviewed the relevant papers and current clinical practice guidelines related to skin cancer (BCC, cSCC and CM) for age-related variations in clinical management and for oncogeriatric aspects of care. We described key aspects of epidemiology, clinical presentation and management for the main skin cancers, BCC, cSCC and CM, in the elderly patient, focusing on potential key-areas for an oncogeriatric intervention.

## Aging population and cancer

The National institute on aging has characterized the aging of our society as a “silver tsunami for which we are unprepared” [10]. Currently, more than 50% of all cancer are diagnosed in patients 65 years and older and this proportion is expected to increase up to 70% by 2030 [11]. Thus, with the progressive aging of the population, geriatric care has become a major issue for health authorities.

Aging is the process of becoming older, a complex scenario that is determined from the interaction of a variety of environmental and genetic factors. This process leads to the loss of functional reserve and increased susceptibility of organ systems. It is linked to the increasing prevalence of chronic diseases and progressive deterioration of organ function, but is also multidimensional and dynamic, as conceptualized by the “frailty” condition of the elderly patient. To quantify aging, it is crucial to differentiate chronologic age from the biological age (or functional aging), in order to detect the multi-dimensional features of this complex process. The normal or successful aging is the focus of Gerontological research, the state of well-being, the condition that can be objectively measured and attained as a positive extreme of the aging process. Thus, by a qualitative approach, the ideal clinical outcome in cancer patients, is to improve the active life expectancy, defined as the average number of years of life remaining in an independent state free from significant disability and with a good quality of life, versus “crude, chronological” life expectancy itself. There are no laboratory or clinical tests that establish the biological age, its evaluation is strictly linked to the functional reserve of systems, which can be efficiently captured by the “Comprehensive Geriatric Assessment” (CGA).

The CGA is a multidimensional assessment tool, in which the different aspects of the elderly population are explored such as comorbidity, functional status, physical performance, cognitive abilities, nutritional status, psychological status, polypharmacy, social support, environmental situation. It is not only a set of questions administered to patients but the entire process of the multi-disciplinary interpretation of the results.

The CGA will unearth unsuspected conditions that are not detected by the standard evaluation and discover reversible conditions that may interfere with cancer treatment, such as multi-morbidity, malnutrition and absence of reliable social support. The CGA is beneficial mainly for patients who are possible to define “frail” [12]. The term “Frailty” is generally used to indicate a state of high vulnerability to negative health related outcomes, such as falls, hospitalization, physical disability, and mortality. The frailty status can be considered as a clinical syndrome, characterized by a combination of a wide range of signs (weakness, fatigue, weight loss, decreased balance, physical inactivity, slowed motor processing and performance, social withdrawal, mild cognitive changes, increased vulnerability to stressors). The prevalence of frailty is usually estimated to be around 10-25% in subjects aged 65 years and older. Even if frailty is closely associated with age, clinical condition and physical impairment, current evidence shows that frailty is predictive of adverse outcomes independently of all these factors [13].

## Skin cancer in the elderly

### Basal cell carcinoma

Basal cell carcinoma (BCC) is the most frequent NMSC in Caucasian population and represents about 80% of all skin cancers [14]. Incidence of BCC continues to rise, increasing by up to 10% over the last decades [15,16]. White individuals of old (65-79 years)-to very old age (>80 years) represent the demographic sub-group with the highest increase in BCC incidence rates [17]. Both genetic and environmental factors may predispose patients to development of BCC. Known risk-factors include male sex, old age, ionizing radiation, immunosuppression, fair skin phototype (Fitzpatrick I or II), chronic arsenic ingestion and family history [18]. Association with intermittent exposure to UV-radiation clearly explain the high incidence rates of BCCs in light-skinned individuals living in countries at low latitudes. Increasing incidence rates are additionally caused by improved screening and diagnosis, increased disease awareness and an aging population. BCC are clinically slow-growing skin tumours, characterized by local tissue invasion and very low rate of metastatic invasion (<0,05%). Nevertheless, BCCs may insidiously invade the underlying structures, causing local tissue destruction, functional impairment, and aesthetic mutilation [19].

BCCs can manifest in different clinical subtypes: superficial, nodular, sclerodermiform, pigmented, and ulcerated. Some of these types are less aggressive (nodular, superficial) than other (sclero-dermiform, ulcus rodens) [20]. In a recent review, Lubeek et al. reported the incidence of BCC among old and very old patients, aged 80 years or more [21]. In this population, incidence of BCC varies depending on skin phototype and geographical location, ranging from 13 to 12,100 per 100000 person/years. BCCs of the elderly are associated with the male gender and located mostly in the head and neck region. Nodular BCC subtype represents the most frequent clinic-pathological subtype. Rarely, inside a BCC lesion, can be found squamous features without clear separation; this mixed morphology tumour has been called basosquamous carcinoma (BSC). The BSC subtype has a more aggressive course and a higher tendency for recurrence and metastases than conventional BCC [22]. The BCC arises from hair follicle stem cells or from progenitor cells in the inter-follicular epidermis. Genetic studies in Gorlin syndrome (or nevoid basal cell carcinoma syndrome), an inherited condition with increased risk of developing BCC, identified germline mutations in the Hedgehog (Hh) signalling pathway. The key component of this pathway is the receptor protein called Smoothened (SMO), which constitutively stimulate cell proliferation, by activating nuclear transcription factors. This signalling pathway is kept in check by a transmembrane protein, Patched (PTCH). BCC carcinogenesis is characterized by aberrant activation of the Hh pathway, resulting from either genetic inactivation of PTCH or activating mutations in SMO [23].

BCCs grow slowly as small erythematous and/or crusted patches or nodules, localized in sun-exposed areas. Early detection and diagnosis by trained dermatologists, with the aid of non-invasive techniques (dermoscopy), are considered key elements for the appropriate management of BCC tumours. “Neglect” of skin cancer is a relevant factor in the elderly, frail patient, especially in the presence of low socio-economic status, functional and cognitive impairment, mood disorders and lack of social support. Delayed diagnosis (in months to years) of BCC can thus lead to extensive, “giant” (>10 cm.) tumours, resulting in local tissue destruction, disfigurement and major surgical defects [24]. Locally advanced BCC comprises tumours not amenable to surgical treatment or radiotherapy due to size, anatomical location in high-risk areas (the “mask area” or central aspect of the face, peri-orificial skin) or patient’s comorbidities [25]. Most of locally advanced BCC and metastatic BCC have been reported in patients older than 55 years, with an estimated prevalence of 0.8% of total case-burden [26]. Despite limited epidemiological evidence due to insufficient data capture, advanced BCC affects a significant number of patients (~29.841 locally advanced cases), causing morbidity and limited survival [27].

## Clinical management of BCC in the elderly

**Figure 1.**
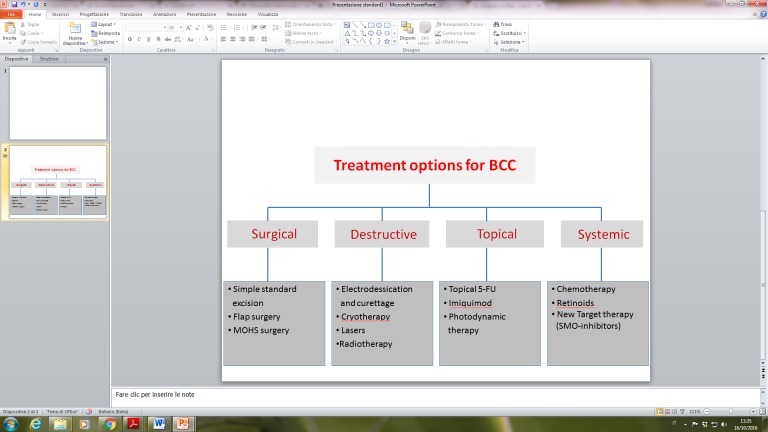
Interventions for Basal cell carcinoma (BCC), by treatment modality.

BCC can be managed with a wide spectrum of treatment options, employing both surgical and non-surgical (medical or physical) modalities. Treatment of BCC is aimed at complete removal of the primary tumour, while minimizing the risk of local spread and maximally preserving the contiguous tissues, thus giving the best functional and cosmetic outcome. The choice of the most appropriate treatment is correlated to the characteristics of the primary lesion as well as to patient’s specific factors [28]. The various treatment modalities are summarized in Figure 1 and include surgery (curettage, conventional excision, Mohs micrographic surgery), radiotherapy and physical treatments (electrodessication and curettage, laser ablation, cryosurgery, photodynamic therapy), medical topical (imiquimod, 5-fluorouracil) and systemic therapies (vismodegib). The current standard of care for BCC is represented by surgery, ranging from standard elliptical excision to complex, micrographic-controlled surgical interventions (Mohs technique) with histological excision-margin control, depending on tumour characteristics, tumour location and regional involvement [29]. BCC area classified in low-risk and high-risk tumours based on risk of recurrence, number of lesions (single or multiple), size, location and clinico-pathological phenotype. High-risk BCC are already relapsed tumours or at risk of recurrence after local treatment/excision, frequently involving the “mask-area” of the face and peri-orificial skin areas. Clinico-pathological phenotypes of BCC also guide treatment decisions, as superficial BCC in low-risk skin areas are easily managed with topical therapies (imiquimod, 5-fluorouracil) and other destructive modalities (cryosurgery, electrodessication with curettage). Nodular, sclerodermiform and infiltrating lesions in high-risk areas should always be treated with surgery, with larger lesions requiring complex defect repairs (skin grafts, flaps and multi-stage surgery), eventually in combination with adjuvant radiation therapy [30].

Cutaneous surgery is generally performed under local anaesthesia and considered a safe procedure, with low rates of morbidity and peri-operative mortality in both in-patient and outpatients care settings. Despite comparable tolerability and safety between younger ad aged individuals, elderly patients with NMSC are frequently excluded from optimal surgical procedures due to pure chronological age [31]. Use of advanced cutaneous surgery, such as Mohs micrographic technique, is controversial in the very old to oldest patients, as these are considered less able to tolerate extensive skin surgery for large tumours and complex plastic reconstructions. Significant variations in the use of micrographic surgery vs. conventional surgery have been reported and related to patient’s age, tumour location and care-settings (outpatient vs. inpatient, private vs, public sector) [32,33]. Rate of peri- and post-operative complications in elderly patients with NMSC varies significantly (0-7.9% up to 20%) in published studies and have been associated with male sex, histology (cSCC), inadequate resection of primary tumour and length of surgical procedure [34,35]. Common complications related to cutaneous surgery include haemorrhagic and infectious ones as well as wound dehiscence and tissue necrosis [36]. Clinical management of locally advanced, inoperable BCC and metastatic disease is challenging due to poor clinical outcomes and limited clinical evidence in current CPGs [37]. Patients with locally advanced and metastatic BCCs are often elderly with multi-morbidity, resulting in significant contraindication to radical-aggressive, multi-stage surgery.

Radiotherapy, in the form of high-energy electron beam, superficial x-rays or superficial brachytherapy, is indicated as second-line treatment for inoperable, primary or recurrent BCC, especially in the case of nodular BCC subtype of the head-neck area (mid-face, nose and eyelids) and in the presence of bone and cartilage invasion [38]. It can achieve good disease control and high cure-rates (90.3% at 5 year), with good cosmetic/functional results and acceptable recurrence rates (7.25-15.8%). Radiotherapy thus represents a useful treatment option in the elderly, frail patients with limited-life expectancy status, especially with more convenient, hypo-fractionated schedules [39]. Adjuvant radiotherapy can be combined with surgery to improve disease control in large, deeply invading lesions with peri-neural invasion [40]. Chemotherapy of locally advanced and metastatic BCC is currently evolving with the recent discovery of targeted therapies, the inhibitors of Hedgehog (Hh)-signalling. Vismodegib was the first oral, small-molecule of this class to receive FDA-approval for the treatment of locally advanced BCC, not adequately managed with surgery or radiotherapy, and metastatic BCC. It determines an effective response rate of 30-43% in patients with uncontrolled local or metastatic disease, which is otherwise a fatal condition with a 8 months-long median survival [41]. Accrual of elderly patients (aged 65 years or more) has been substantial (>62%) in clinical studies and expanded access programs with vismodegib treatment. Common class-related adverse events during vismodegib therapy, such as muscle spasm, dysgeusia, weight-loss and fatigue, are mild-to-moderate and run a chronic course, impacting quality of life and leading to treatment discontinuation in almost one third of patients [42]. An accurate oncogeriatric assessment of the elderly patient could potentially support adherence to treatment in elderly patients with advanced BCC. Current, EADO/EDF and NCNN, clinical practice guidelines (CPG) for BCC do not systematically address oncogeriatric factors in the elderly, frail patient subgroup [30,43]. In the “real-life” clinical practice, treatment decisions are mostly based on tumour characteristics and physician’s preference, expertise and operational setting, while age, comorbidities and patient’s preference are frequently overlooked.

Only pure “chronological “age and patient’s comorbidities, especially immunosuppression, are considered relevant patient-centered factors. In a recent survey and consensus by a multidisciplinary expert group of dermatologists and onco-geriatricians, “limited life-expectancy”, comorbidities and “treatment goals other than curation” were identified as key items for onco-geriatric care of NMSC in frail, older patients [44]. Other common oncogeriatric domains, such as frailty, geriatric syndromes, nutritional, functional and mood status, social support are not routinely evaluated in the clinical practice, as the majority of NMSC are not considered associated with risk of dissemination and mortality in the adult population. Furthermore, on BCC disease spectrum, the management of low-risk tumours in old, frail patients or old patients with limited life-expectancy can prove challenging, eventually leading to significant over-treatment [45].

A recent, prospective observational study examined the impact of limited-life expectancy on the treatment of NMSC in a large, elderly patient cohort (n=1536). In the study, more than half of NMSCs were treated surgically, irrespective of concomitant LLE status in treated patients. LLE status did not affect treatment patterns in BCC, as wells as for cSCC, even when controlling for patient, tumour and care setting characteristics. Almost half of the patients with LLE status died within 5 years, with no NMSC-related death case. In the LLE group, 20% of patients were at risk of treatment-related complications of NMSC, such as poor wound-healing, bleeding, infection and pain symptoms after surgical treatment [46]. In the routine clinical care of BCC (and other NMSC), LLE status and other relevant patient-based factors (comorbidities, functional status) do not significantly guide treatment decisions in elderly patients, with the risk of reducing the overall clinical benefit especially for patients with a low-tumour burden. A simplified, clinical approach to the management of low-risk BCC in patients with LLE has been recently proposed, but currently lacking clinical validation and integration with current CPGs [47]. Mohs micrographic surgery represents the gold standard for the treatment of NMSC in the head and facial areas, especially for high-risk tumours. This treatment modality could prove problematic for elderly, frail patients, which poorly tolerate long surgical procedures (mean duration of surgery 3 hours) and require assistance for activities of daily living (ADL) as well as for wound-healing practices and follow-up after surgery. Few studies have analysed the impact of complex Mohs micrographic surgery modalities in clinical outcomes of old to very-old patients with NMSC. In a retrospective study on 214 nonagenarians, surgical treatment of both BCC and cSCC with Mohs micrographic surgery did not affect survival, with no increase in perioperative mortality and morbidity [48]. In this study, specific cause of death was not recorded, but tumour-related factors and complexity of the surgical procedure (defect-size, number of surgical stages, closure type) did not change life-expectancy. In another prospective study on clinical outcomes of old patients (aged 80 years or more) after cutaneous surgery for NMSC, increased mortality was associated with age, Charlson comorbidity index, Barthel index and closure of the surgical defect with a skin graft. Common causes of death included advanced neurologic disease and cardio-vascular disease [49]. Management of both low-risk and high risk BCC in the frail, old to very-old patient, at the end of life, thus remains challenging, as there is limited clinical evidence and a guidance to support rational and patient-centered treatment decisions. Furthermore, implementation of appropriate clinical tools for the onco-geriatric screening and assessment of patients in the busy, high-volume dermatological care setting is still missing. The Charlson comorbidity index has been indeed proposed as a generic tool for risk-stratification of old patients with NMSC candidate to surgery [50]. Future clinical validation and feasibility studies should evaluate the role of oncogeriatric screening and assessment tools in elderly patients with NMSC.

## Cutaneous squamous cell carcinoma

Cutaneous squamous cell carcinoma (cSCC) is a typical tumour of elderly individuals, resulting from the malignant transformation of keratinocytes of the epidermis and its appendages. It is a typical tumour of advanced age (mean age of 70 years at diagnosis), with more than 80% of cases occurring in old patients. Invasive cSCC represents roughly 20% of NMSC, developing de-novo on chronic sun-exposed skin or from precursor lesions, the in-situ, intraepidermal cSCC (Bowen’s disease) or actinic keratosis (AK). cSCC arises typically on chronic sun-exposed skin areas, involving the head and neck area as well as the dorsal aspects of the upper limb area in almost 90% of cases [51]. Epidemiological data on cSCC incidence, mortality and disease burden is limited and fragmented, as these tumours are excluded from national cancer registries. Moreover, there is a potential overlap of in case-definitions of cSCC with the broad category of head-and neck cancers. In most epidemiological studies on cSCC, tumours of lip vermillion area and ano-genital cSCC area excluded from analysis. Studies from different western-industrialized nations have reported a dramatic increase (50-200%) in incidence rates of cSCC during the past three decades, reflecting an increase of cumulative UV-exposure and and an aging population [52]. In the United States (2012), estimated cSCC cases in the white population ranged from 186,157 to 419,843, with nodal disease involving between 5604 and 12,572 persons, depending on latitude and UV index [53]. Age-standardized incidence rates for cSCC present a marked geographic variability, ranging from 30 per 100.000 persons/year in Germany to 1332 per 100.000 persons/year in Australia [51]. Disease-specific mortality of non-genital cSCC is generally considered to be low (1.5-2.1% estimated risk) and declining in several western countries, but there are several areas of uncertainty related to misclassification of disease coding. Recent estimates report between 3932 to 8791 cSCC-related deaths in the United States, with regional figures in central and southern states exceeding mortality from melanoma, renal cell and oro-pharyngeal carcinoma [52]. Incidence of cSCC increases dramatically with age, as cumulative, chronic (occupational or recreational) sun exposure is the main risk factor for development for this keratinocytic tumours [54]. Other predisposing factors include exposure to ionizing radiation, toxic chemicals (arsenic acid, polycyclic hydrocarbons) and very long-lasting cutaneous inflammation associated with chronic wounds, ulcers, radiodermitis, old burn and scars [55]. Immune suppression, as observed in Organ transplant recipients (OTRs) and during treatment of haematogical conditions, is another strong risk factors for cSCC, influencing also disease progression and course [56].

Unlike to BCC, invasive, high-risk cSCC can spread to loco-regional lymph-nodes and then to distant sites as metastatic disease. The risk of metastasis in cSCC is variable from 3 to 16.4% depending from intrinsic tumour prognostic factors and up to 20%, in special patient subgroups, such as OTRs [57-59]. Metastatic disease involves loco-regional lymph nodes in almost 85% of cases, followed by distant visceral metastasis (lungs, bone, liver, brain). Disease staging of invasive cSCC follows the TNM system of the AJCC and UICC, which have been criticized due to incomplete validation and major overlap with the more heterogenous group of conventional head-and neck cancers [60]. Alternative staging systems for cSCC have been proposed, to differentiate low- vs. high risk tumours in terms of clinical outcomes and prognosis [61]. High-risk cSCC defines a subset of tumours with aggressive biology, presenting a significant risk of local recurrence after surgery (10-47.2%) and of regional and distant metastatic dissemination (11-47.3%). High-risk features of cSCC include mainly a combination (of at least two) pathological tumour characteristics, such as tumour size (>2 cm.), tumour depth (>2 mm.), tumour site (lip, ear), poorly differentiated histology, histologic type (spindle, desmoplastic, acantholytic), perineural or lymphovascular invasion, recurrent or rapidly growing tumours. Patient-based factors for high-risk disease include only chronic immunosuppression (OTRs and chronic lymphocytic leukemia). High-risk cSCC is associated with lower three-year disease specific survival (70%) than low-risk tumours, with lower survival rates (30% at 5 years) and higher recurrence rates in nodal disease and in OTR patients [62].

## Clinical management of cSCC in the elderly:

**Figure 2.**
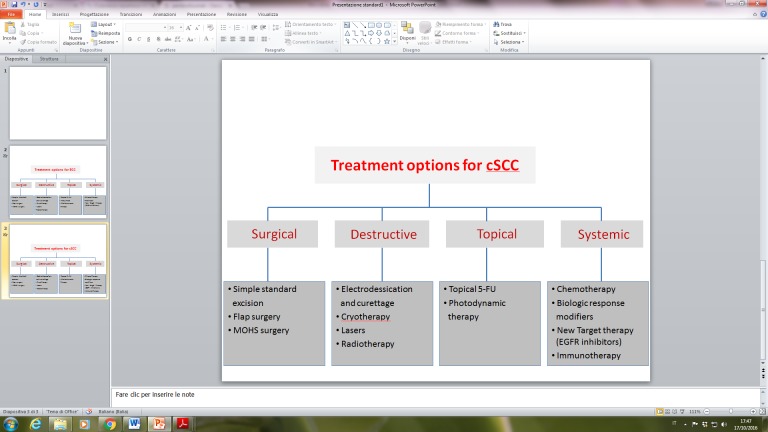
Interventions for cutaneous squamous cell carcinoma (cSCC), by treatment modality

Current standard of care for cSCC is described in details in the European (EADO/EDF) and American (NCNN) guidelines, with treatment options summarized in Figure 2 [63,64]. As in BCC, complete surgical excision with histopathological control of excision margins represent the gold-standard of treatment of primary invasive cSCC. Standard surgical excision and Mohs micrographic surgery and its variants determine optimal disease control in more than 90% of cases, while preserving normal tissue function and adequate cosmetic results in known danger zones (lips, periorificial areas, nose and ears) [65]. Limitations for radical surgical treatment can occur in very large (>2 cm. in diameter) and thick (> 6 mm. in thickness) invasive tumours, with high-risk characteristics, in order to guarantee adequate excision margins (6-10 mm.). Multiple and recurrent cSCCs affecting the head-and neck area can also pose some technical difficulties for surgical treatment and require extensive surgery and plastic reconstruction [66]. Patient’s age, comorbidities, functional status and concomitant medication can negatively impact indication to surgery. Inoperable cSCCs due to patient-based factors or locally advanced tumours can be treated with radiotherapy, either as an elective or as an adjuvant treatment option [38].

Selection of appropriate treatment for advanced, high-risk cSCC can be challenging in frail, elderly patient with LLE status. As in general for NMSC, there are only few clinical studies addressing the impact of geriatric assessment and related factors on treatment decisions and outcomes for cSCC. As discussed previously, LLE status in elderly patients is not considered as relevant factor for indication to surgical treatment of NMSC, despite the increased risk of adverse events in this group of patients [46]. In the European guideline, a general recommendation to evaluate patients’ age and life-expectancy is made to guide patient selection and indication to radiotherapy, minimizing risk of secondary malignancies [63]. Clinical management of locally advanced and metastatic cSCC, especially in the elderly patient, is another relevant practice gap, due to limited clinical evidence. Locally advanced tumours, inoperable tumour recurrences and in-transit metastasis of cSCC are treated with radiotherapy, electrochemotherapy or chemotherapy, aiming at disease control or palliation [67]. Electrochemotherapy is an experimental treatment modality combining high-dose bleomycin and cisplatin with electrical-mediated cell membrane permeability. It is a complex, inpatient-based procedure with a substantial efficacy and good safety profile in highly-selected cases of NMSCs [68].

## Cutaneous Melanoma of the elderly

The elderly population is at the highest risk to develop cutaneous melanoma (CM).

In recent decades, the global epidemiology of melanoma presented a continuous increase in incidence rates and divergent, stable mortality rates, confirming the notion of a melanoma epidemic in western-industrialized countries [69]. In the United States and Australia, the highest rate of increase in melanoma incidence have been consistently observed in the elderly white-male population (aged 60 years) and in lower socio-economic areas. In the United States, more than 40% of melanomas involve patients older than 65 years, resulting in 60.2% of overall melanoma-related mortality. Whereas favourable trends in melanoma-related mortality have been reported in young adults and women during the last two decades, the old age patient subgroup (>65 years) still presents an increasing trend in mortality [52]. These findings underline a greater burden of disease in the elderly population, resulting in significant economic impact, with estimated annual costs of 249 million dollars and per-patient lifetime costs (~$28 210), which are mainly related to late-stage disease [70]. Together with male sex, age is one of the most important prognostic factor for melanoma patients [71]. Furthermore, melanoma of the elderly presents distinct features in clinical presentation and disease-course in comparison to melanoma in young adults, suggesting divergent biological and molecular profiles. [72]. Poor clinical outcomes among elderly patients with melanoma can be also explained by additional factors, such as a dysfunctional immune response, the impact of frailty syndrome and comorbidities as well as adverse variations in clinical management [73].

Early diagnosis and secondary prevention of MM are limited in the elderly population, due to difficulties in self-skin examination, low-disease awareness, neglect and low socio-economic status. Delayed diagnosis frequently leads to primary tumours in advanced stages, with thick, ulcerated, rapidly growing, invasive lesions and consequently poor clinical outcome.

In primary cutaneous melanoma, increasing age is associated with increasing Breslow tumour thickness, the most important prognostic factor related to tumour stage and survival [74]. Prevalence of thick melanomas increases up to 20% at the age of 80 years, especially in male patients. Older patients present a higher proportion of tumours with head-neck localization, greater mean Breslow tumour thickness, higher frequency of adverse histologic markers (ulceration, high mitotic index), resulting in more advanced tumour stages at diagnosis than in younger patients [75]. Elderly patients have thus consequently up to 10% lower disease-specific survival rates compared to younger ones [76].

The main clinico-pathological variants of melanoma are classified as superficial spreading melanoma, nodular melanoma, lentigo maligna-melanoma and acral lentiginous melanoma, each with well described biological and clinical features. The clinico-pathological patterning of melanoma is slightly different in the elderly patients, with increased prevalence of melanoma associated with chronic sun damage and nodular melanoma [75]. Superficial spreading melanoma is the most common type of primary cutaneous melanoma in the young adult population, but becomes less frequent (~31.6%) with advancing age. It typically involves intermittently sun-exposed skin areas, displaying a signature molecular profile associated with BRAF-mutations. Melanoma associated with chronic sun-damage affects the skin of the head-and neck areas, face and dorsal-distal aspects of the extremities, in combination with other clinical signs of chronic, cumulative sun exposure (solar elastosis, lentigines and actinic keratosis). This melanoma sub-group presents a variable molecular profile, with KIT and NRAS gene mutations, with an initially more radial, intraepidermal (in-situ) growth pattern [77].

Lentigo maligna-melanoma (LMM) is the prototypical, chronic sun-damage associated variant in the elderly patient, with a mean age of diagnosis of 65 years, accounting for 11.1-24.2% of diagnosed subtypes in this age group. Lentigo maligna (LM) represent the intra-epidermal, in-situ stage of disease, before progressing to frank, invasive LMM tumour. LM presents as large, irregular pigmented macule or patch on sun-exposed, visible skin areas, such as the face, with a slow, indolent radial expansion over a period of years. Acral lentiginous melanoma is rare variant (1-2%), involving the glabrous palmo-plantar skin, and is not associated with chronic sun-damage. If left undiagnosed and un-treated, lentigo-maligna and superficial spreading melanoma variants progress over a variable time-period (years) from in situ-stages, to frank, invasive tumours, switching to a vertical growth phase, with the risk of loco-regional (lymphatic) or systemic (hematogenous) dissemination. Nodular melanoma is on the other hand an aggressive clinico-pathological variant, presenting as rapidly growing nodular lesion in the vertical, invasive growth phase, with high risk of loco-regional and metastatic spread. Nodular melanoma is more frequent in aged individuals, representing 15%-33.9% of diagnosed melanomas old to very old patients (>85 years) and thus a negative prognostic factor. Despite the impact of late diagnosis, socio-economic status and comorbidities and clinical prognostic factors, old age still represent an independent factor negatively affecting overall survival in melanoma [78].

In fact, old age seems to modulate melanoma progression, as invasive tumours appear to disseminate preferentially via a haematogenous route rather than via lymphatic spread. Sentinel lymphnode biopsy (SLNB) is a fundamental disease staging method to assess the status of regional lymphnode basin and represent one the most significant prognostic factor for melanoma. The rate of sentinel-lymphnode involvement decreases with increasing age, resulting in a lower risk of SLN metastasis in older individuals (>60 years) compared to young adults, independently of tumour characteristics [79-81]. The decreased rate of lymphatic dissemination in elderly patients is not associated with an improved survival, but could reflect an inherently different biology of melanoma or dysfunctional lymphatics in the old patient. Age-related decline in lymphatic function has been demonstrated to reduce both afferent transit and uptake of the radiocolloid to the regional sentinel lymph node, thus modifying metastatic patterns in elderly patients [82]. Anatomical changes of the dermis, lymphatics and lymphnodes related to aging can possibly determine lymphatic dysfunction, favouring loco-regional and in-transit spread of tumour cells. Recent experimental data support indeed the notion of distinct tumour progression in the old individuals, due to an aged, dermal tissue microenvironment favouring melanoma cell invasion and metastasis [83]. “Immuno-senescence”, defined as the age-related decrease in immunological competence, may be another critical factor, negatively impacting clinical outcomes of melanoma in elderly individuals. The progressive impairment of both innate and adaptive immunity affects responses to pathogens, and vaccines as wells as anti-tumour surveillance [84]. Evidence of an aging-related T-cell dysfunction has been recently reported in gene-expression studies of different melanoma-patient cohorts [85]. Tumour-infiltrating lymphocytes (TILs) in primary melanoma represent a known pathological, albeit surrogate, marker of the host anti-tumour immune response. In middle-aged (>45 years) and old-aged (65 years) individuals, intensity of TILs seems to carry a greater prognostic significance than in younger melanoma patients. Reduced or absent TILs in elderly patients have been associated with an increased risk of disease recurrence and worse melanoma-specific outcomes [86].

Elderly patients with CM thus presents unfavourable patterns of clinical and prognostic factors, resulting from a complex interplay between a distinct tumour biology, host-tumour interactions and age-related variations in diagnosis and clinical management. Sub-optimal clinical management and under-treatment of melanoma in the elderly population remains an un-resolved issue in dermato-oncology, despite established CPGs. Old age is in fact associated with significant variations in clinical management of CM, for both primary, localized tumours and loco-regional-metastatic disease [87]. This could be a potential confounding factor for the interpretation of poor disease-specific outcomes in this specific patient group [88]. Age-related variations in clinical management of CM, across all disease stages, have been reported in observational and population-based epidemiological studies. Old to very old patients with CM have thus limited access to optimal surgical treatment and staging techniques, such as Sentinel Lymphnode Biopsy (SLNB), in comparison to younger ones [89].

## Clinical management of cutaneous melanoma in the elderly:

In melanoma, current CPGs consider patient age and overall performance status (ECOG) as a relevant factor for guiding clinical management and treatment decisions in elderly patients [90,91]]. In the elderly patient, dermato-oncologists are frequently confronted with several clinical scenarios across all different stages of MM, which could clearly benefit from an integrated, oncogeriatric approach. Despite recommendations in the CPGs, few observational or interventional studies have reported the impact of oncogeriatric factors in the clinical management of primary cutaneous melanoma [92]. Furthermore, the treatment landscape of loco-regional (stage III) and advanced, metastatic disease (stage IV) is rapidly changing, due to recent advancements in selective, molecular-targeted treatment strategies and in immunotherapy of melanoma. Key-areas for an oncogeriatric intervention in the clinical management of melanoma are summarized in Table 3.

**Table 3:** Key areas for oncogeriatric intervention in the clinical management of cutaneous melanoma.

Treatment decision	Rational for oncogeriatric evaluation and intervention
Excision of primary tumour	Excision margins depend on tumour thickness Insufficient excision margins correlated with old-age Indication for micrographic excision surgery for LMM
Non-invasive treatment for in-situ LM	Topical immune-modulators as alternative to conventional surgery in selected patients
Sentinel lymph-node biopsy (SLNB)	Indication to the staging procedure may be influenced by patients’ characteristics and decreased rate of SLNB positivity with increasing age
Complete lymph-node dissection (CLND)	Indication to CLND limited by risk of morbidity and complications in the old patient; less performed in old age
Adjuvant therapy	Risk-benefit analysis of interferon-alpha treatment or other investigational immunotherapies in the old patient, with LLE status
Surgery of distant metastasis	Selection of fit vs. frail patient for surgery to improve overall survival
Immunotherapy of metastatic disease	Inclusion of old, very old and oldest patients in clinical trials and expanded access programs; improved prevention, surveillance and management of irAEs
Targeted treatment/chemotherapy of metastatic disease	Inclusion of old, very old and oldest patients; identification of pre-frail patients at increased risk of AEs

(LM= Lentigo maligna; LMM=Lentigo maligna melanoma; LLE=Limited Life Expectancy; irAEs=immune-related adverse events; AE=adverse events)

In primary localized disease, the accurate diagnosis and identification of distinct clinico-pathological variants of melanoma is a first step to guide treatment decisions. Non-invasive diagnostic techniques, such as dermoscopy and reflectance confocal microscopy (RCM), are often employed by trained onco-dermatologists to diagnose melanoma with high accuracy (90-95%), differentiating melanoma subtypes and improving clinico-histological correlations [93].

The in-situ, lentigo maligna (LM) variant, is amenable to both conservative surgical management and non-invasive, alternative treatment methods, such as topical imiquimod treatment [94]. Imiquimod, as described previously, is a potent TLR-7 agonist and several clinical studies support its use as an alternative option to surgery in selected cases of LM. Patient’s age, comorbidities and the presence of contraindications to surgery are primary factors in guiding this treatment decision. An integrated oncogeriatric assessment is this relevant in this case to support and validate treatment decisions of LM in the elderly patient. This is crucial aspect, as imiquimod treatment presents a 76% histological and a 78% clinical clearance rate of LM, resulting in a discrete risk of local recurrence (~3%) [95]. Treatment of localized/primary CM is eminently surgical and old age is associated with significant variations in surgical management. In primary localized disease (stage I-II), surgical management is frequently sub-optimal in the elderly patient, presenting inadequate excision margins and subsequent higher-risk of local recurrence, independently from known prognostic factors [89]. Furthermore, an increasing burden of comorbid conditions, as assessed by the Charlson comorbidity index, has a negative impact on disease-specific and overall survival in the elderly melanoma patient [96].

Indication to SLNB procedure is another challenging clinical scenario in the old- to very old patient, with intermediate risk melanoma (tumour thickness >1.0 mm, stage IB-II disease), due to the supposedly decreased prevalence and significance of SLNB positivity in elderly patients. SLNB is a widely accepted staging technique in intermediate-thickness melanoma and is a convenient, minimally invasive procedure, which can be performed under general anaesthesia or in the outpatient setting, at the time of wide local excision for the primary tumour. As of now, SLNB status is a critical step in melanoma-staging also in the elderly patient population, and useful for identifying patients at high-risk of progression qualifying for completion lymphnode dissection (CLND), adjuvant treatment or for potential enrolment in clinical trials [97]. In current CPGs, decision not to perform SLNB may be based on significant patient comorbidities, patient preference of other unspecified factors. In the elderly, frail patient, with LLE status, the option to perform an SLNB for low- to intermediate risk, clinically node-negative MM should be carefully discussed, weighing the benefits of an accurate tumour staging with potential risk of morbidity of the procedure [98]. The rate of SLNB utilization in elderly patients is highly variable (23.3-82%), depending from study designs (registry- vs. single-center based studies), operational settings (secondary care vs. tertiary referral skin cancer centers) and healthcare systems-related factors [89, 99]. In single-centre based retrospective studies, decision to perform a SLNB in eligible, elderly melanoma patients is mainly affected by pure “chronological” age, performance status, tumour location (head and neck primaries), surgeon’s and patient’s preference, whereas the impact of medical comorbidities (CCI index) appear to be limited [100, 101]. When a SLNB is positive (micro-or macro-metastasis), an immediate or delayed completion lymph node dissection (CLND) is usually performed. Immediate CLND is indicated in patients with positive SLNB to improve disease control and overall survival. The rate of CLND after a positive SLNB is reportedly lower in older patients (aged >70 years), with a decreased surgical yield of lymph nodes, according to population- and registry-based studies [102-104]. The therapeutic value of CLND, aimed at improving quality-adjusted life expectancy (QALE), could prove limited in frail, aged individuals by long-term, surgical complications (lymphedema, nerve damage and wound complications) [105]. This clinical scenario represents a potential area for an oncogeriatric intervention, evaluating relevant patient-based factors and balancing the decision between CLND vs. observation. In a single-centre, retrospective cohort study, sarcopenia, a known objective marker of biological frailty in the elderly, has been reported to be a relevant predictor of clinical and surgical outcomes among elderly patients with stage III melanoma undergoing CLND. In this patient cohort, decreased disease-free survival and distant disease-free survival as well as a higher rate of CLND-related surgical complications were significantly associated with sarcopenia, independently from age [106]. The management of regional lymphnode with SLNB or CLND in the elderly melanoma patient thus presents several areas of uncertainty, especially in the very-old, frail individuals with limited life-expectancy (LLE) and at risk for short-term and long-term surgical morbidity.

Treatment of high-risk melanoma, loco-regional disease (stage III) and metastatic disease (stage IV) is an evolving scenario in dermato-oncology, due to significant breakthrough in molecular targeted therapy and immunotherapy in the last decade. Treatment options for advanced loco-regional disease and metastatic disease are rapidly expanding, combining surgery, intralesional/regional therapy, systemic therapies and radiotherapy, to improve for the first-time disease-free survival and overall survival of melanoma patients. Adjuvant treatment is currently proposed to patients after complete surgical resection of stage III melanoma at high-risk of recurrence, to prevent disease progression. Older melanoma patients (aged >70 years), at high risk of recurrence, are disproportionately excluded from adjuvant treatment, systemic treatments and participation in clinical trials than young adults, contributing to worse clinical outcomes [107].

Currently, the adjuvant treatment setting includes mainly high-or low-dose interferon-alpha regimens and other investigational therapies, such as immune checkpoint inhibitors.

In patients with resected stage III melanoma at high risk of recurrence, high- or low-dose adjuvant inteferon-alpha-2b regimens have been demonstrated to improve only disease-free survival, at the cost of dose-dependent adverse events [108]. In population-based studies, adjuvant interferon treatment is less frequently proposed to older patients than in younger ones (18.9% vs. 58.8%) [89,107]. In older patients, interferon therapy was more frequently interrupted compared to younger ones (73.3% vs. 34.1%), due to disease progression, poor tolerability (50%) and compliance [89]. In very old individuals (>75 years) and patients with LLE status and comorbidities, indication to adjuvant interferon should be carefully discussed, highlighting the role of a comprehensive geriatric assessment in guiding the benefit/risk analysis and treatment decisions. Furthermore, an oncogeriatric approach to the old, frail patient with resected high-risk, stage III melanoma will be of increasing value, as the role of the new immune checkpoint inhibitors (ipilimumab) has been recently explored for long-term, adjuvant treatment [109].

Immune checkpoint inhibitors (ICI) represent a breakthrough in oncology, improving progression-free and overall survival in metastatic melanoma and other cancer types (non-small cell lung cancer, renal cell cancer, bladder cancer, head-and neck squamous cell carcinomas). ICI include two main groups of monoclonal antibodies, ipilimumab, targeting the CTLA-4 (cytotoxic T-lymphocyte antigen-4), and the PD-1 inhibitors, with nivolumab and pembrolizumab targeting the programmed-death-1 antigen. Both types of ICI modulate the activation of T-cells by blocking inhibitory signals in the priming or in the effector phase of the immune response, restoring an effective immune response against tumour cells. Immunotherapy of metastatic melanoma with single agent (nivolumab, pembrolizumab) or double agent (ipilimumab and nivolumab) checkpoint inhibition determined a significant survival benefit and durable responses in clinical trials, showing a better safety profile in comparison to conventional chemotherapy [110]. The safety profile of ICI is characterized by immune-related adverse events (irAEs), a new class of inflammatory and autoimmune toxicities, caused by infiltration of autoreactive T-cells in normal tissues. Severe irAEs are infrequent (~10%) in published studies, but can lead to discontinuation of immunotherapy and even to life-threatening conditions. The risk of severe irAEs and the phenomenon of immune-senescence could pose a significant concern on safety and efficacy of ICI in the older adults, especially in the presence of the frailty condition and decreased performance status. Accrual of elderly patients in clinical trials and expanded-access programs of melanoma immunotherapy has been substantial, with an average of 38% of old (>65 years) melanoma patients [111]. In preliminary, retrospective analysis of old patient subgroups, there were no significant differences in terms of efficacy and safety of ICI in comparison to young adults [112, 113]. Also, the incidence and clinical spectrum of irAEs appears to be similar between younger and older adults, especially in the 65 to 75 years’ age-group. In the very old patient group (aged >80 years), preliminary evidence points to an increased trend of irAEs, leading to treatment discontinuation and co-medication with steroids and immunosuppressive drugs [114]. The clinical impact of irAEs has been reported to be significant in the very old patients treated with dual checkpoint inhibition. Immunotherapy of metastatic melanoma remains an important treatment option in the elderly patient population and real-life clinical data will hopefully shed light on its efficacy and safety. An oncogeriatric intervention approach could potentially improve clinical management also in this scenario.

## Quality of life issues in the elderly skin cancer patient

The CGA can assess the needs of the patients, the degree of self-sufficiency, the biological age compared chronological age, physical and cognitive performance, but what may be more beneficial in assessing skin cancer patients? We believe that considering the new treatments and the progressive increase of disease-specific outcomes, probably the most important parameters in the assessment of elderly patients with skin cancer is quality of life in combination with life expectancy. The term “quality of life” encompasses the subjective state and capability to act in the physical, mental and social realm. It is the perception of the effects of illness and treatment on the physical, psychological and social aspects of life [115]. In the last 20 years’ patient reported Quality of life (QoL) assessment has become an increasingly important factor in the global assessment of many disease, including cancer [116]. Moreover, the impact of cancer on Health-related quality of life (HRQOL) is poorly understood because of the lack of baseline HRQOL status before cancer diagnosis, or because it is not compared to individuals without cancer. The impact of cancer is often estimated in terms of clinical endpoints such as the risk of recurrence and the probability of remission and survival; but these measures don’t fully capture the impact of cancer in terms of its effect on a person’s functioning and well-being. It’s not easy make studies on elderly oncological patients based on HRQOL, in particular among those with other chronic conditions that likely also affect HRQOL [117]. So, to assess changes in HRQOL that are mainly attributable to the cancer and that are less likely related to other potentially confounding characteristic, pre-diagnosis assessments and comparisons of cancer patients with appropriate control groups are need [118,119]. A study examined the impact of a new cancer diagnosis on HRQOL among older Americans, in 2009 was the first study to report HRQOL changes from before to after cancer diagnosis across most cancer site [120]. The assessment of quality of life constitutes an important endpoint in health services research. It has been established in general oncology, while it has only recently become a focus of interest in dermatologic oncology [121,122]. The impact of NMSC on patients may arise from the tumor itself or as a result of treatment, and trough symptoms, functional limitations, cosmetic burden and auxiliary considerations such as cost and disturbance to the activities of daily living. Many NMSC appear on the face or other visible areas of the skin, it could be symptomatic, with bleeding, pain and pruritus [123]. Moreover, most NMSC are treated with surgery, interrupting the normal activities of daily living, and have a financial impact, and repeated treatments may be needed in the setting of incomplete surgical margins or recurrence. After treatment, there are cosmetic and functional sequelae from scarring that can affect psychosocial function, this burden was assessed using a variety of outcome measures.

Standardized, validated questionnaires to assess quality of life in cancer patients such as, SF-36, PLC or EORTC-QLQ-C30, are widely used [124-126]. Those instruments are generic, not focus on elderly and with no evidence in literature about non-Melanoma Skin Cancer. Several studies investigated the burden of NMSC on QoL with those instruments, but they proved a QoL similar to those expected in the normal population thus the authors attributed the results to the insensitivity of the instruments and recognized the need for a disease-specific instrument. Rhee’s group was the first to recognize the need for a comprehensive disease-specific instrument to adequately capture the impact of NMSC on QoL, so a group of specialists (dermatologist, facial plastic surgeon, oculoplastic surgeon and plastic surgeon) proceeded to develop the Skin Cancer Index (SCI). The tool does not address patient’s concern such as prevention, follow-up/early detection and guilt. This study had the objective to create, validate and test a specific Skin Cancer Quality of Life. The final SCQoL is a 9 item Rash derived scale and consists of nine item; the score can also be reported as a sub scores on three domains covering function (item 1, 8, 9), emotions (item 2, 4, 6) and control (item 5, 7); item 3 is considered as a global item [127]. Limitations are the cultural bias due to the monoculture development of the tool as wells as the discrepancy between patient’s self-assessment and physician’s assessment. Most of the questionnaires shared this potential limitation. Korner et al. have tried to examine the relationship between patients’ needs and distress, general and skin-cancer specific and to assess the prevalence of skin cancer specific distress, general distress and the supportive care needs. The innovation introduced in this study is the use as distress assessment the Hospital Anxiety and Depression Scale (HADS) [128]. The Dermatology Life Quality Index (DLQI) is probably the most frequently dermatology-specific patient-reported outcome measure (PROM) for quality of life [129]. It consists of ten questions that illuminate the perception of the patient respect to his skin disease regarding the time-period of the previous seven days; the effects of the skin disease on feelings, daily activities, work or school, personal relationships and treatment side effect are inquired. Each question is to be answered on a 4-point scale: 0 (not at all) - 3 (very much). Every individual score values are added up to a total score that can range from 0 to 30; higher scores denote a greater impairment of the quality of life. NMSC does not seem to impact greatly on the QoL patients, but keep looking for more and better assessment to make sure this is so it’s necessary. Recognizing patient need, cancer-related concerns and early signs of distress is the first step toward addressing them. To provide efficient PROMs for patient’s QoL is essential for a proper cancer treatment, especially for elderly patients, who usually do not talk directly about their concerns, often with economic limitations, difficulty taking part in social activities, physically and emotionally unstable, liable to feel lonely [130-132]. In today’s aging society, multidisciplinary intervention and training for healthcare professionals will be required to deal with different and complex concerns of elderly patients with cancer. Also in the case of skin cancer and related interventions, clinicians should make an active effort to consider potential concerns of elderly cancer patients who do not complain, predict their possible problems such as upset and intervene on them. Combined efforts from dermatologists and gerontologist, with the aid of a multi-disciplinary team, can thus deliver optimal patient-oriented, oncological care, improving patient’s quality of life and adherence to treatment and follow-up programs.

## Conclusions

A major limitation for an oncogeriatric approach in Dermato-oncology is the lack of validated and optimized clinical tools for the screening and comprehensive assessment of the elderly patient. In current CPGs, there is no guidance or specific evidence to support one or more clinical tools for the oncogeriatric screening or assessment of the elderly patients with NMSC. Onco-geriatric patient evaluation should ideally focus on rapid, easy to administer and validated clinical tools, to adapt to the real-life conditions of dermatological care settings, which are often characterized by high-patient numbers and limited consultation-time. This is especially relevant for the care of NMSC patients, which are a highly heterogeneous patient group, presenting a wide disease spectrum, in terms of morbidity and low disease-specific mortality [133]. In the case of head-and neck cancers, oncogeriatric screening tools (G-8, VES-13) followed by the comprehensive geriatric assessment (CGA) has been used to screen and prospectively evaluate vulnerable patients during radiotherapy [134]. In the elderly, frail patient with high-risk NMSC, future prospective studies should ideally evaluate the impact of an oncogeriatric intervention on treatment decisions, selected clinical outcomes and relevant patient-reported outcomes. In cutaneous melanoma, age-related inequalities in the clinical management could be potentially overcome with a more accurate, interdisciplinary clinical assessment of the elderly patient, effectively differentiating “fit” from “frail” patients. Current CPGs do recommend an oncogeriatric intervention mainly in the setting of advanced, metastatic disease, but, as discussed previously, several practice gaps persist in the clinical management of primary tumours and high-risk loco-regional disease. Since the number of elderly patients with CM will greatly expand in the near future, the clinical need for equal and effective surgical and medical management will likewise increase. Future clinical studies should ideally target the elderly population with CM at high-risk of recurrence as well as with metastatic disease, elucidating the interaction between immunotherapy and the aged immune system. In conclusion, a geriatric and patient-based treatment approach in dermato-oncology could be valuable for stratifying the elderly patient with skin cancer across all available treatment options, optimizing treatment outcomes, quality of life and compliance, while addressing the socio-economic aspects of cancer care.
